# Vasohibin 2 reduces chemosensitivity to gemcitabine in pancreatic cancer cells via Jun proto-oncogene dependent transactivation of ribonucleotide reductase regulatory subunit M2

**DOI:** 10.1186/s12943-017-0619-6

**Published:** 2017-03-21

**Authors:** Min Tu, Haifeng Li, Nan Lv, Chunhua Xi, Zipeng Lu, Jishu Wei, Jianmin Chen, Feng Guo, Kuirong Jiang, Guoxin Song, Wentao Gao, Yi Miao

**Affiliations:** 1 Pancreas Center, The First Affiliated Hospital with Nanjing Medical University, Nanjing, 210029 People’s Republic of China; 2 Department of Pathology, The First Affiliated Hospital with Nanjing Medical University, Nanjing, China

**Keywords:** Pancreatic cancer, Vasohibin 2, Gemcitabine, Ribonucleotide reductase regulatory subunit M2, Jun proto-oncogene

## Abstract

**Background:**

Vasohibin 2 (VASH2) has previously been identified as an agiogenenic factor and a cancer related protein. Here we investigated the association of VASH2 expression and chemoresistance in pancreatic cancer.

**Methods:**

Immunohistochemical staining for VASH2 was performed on 102 human pancreatic cancer samples. Pancreatic cancer cell line models exhibiting overexpression or knockdown of VASH2 were generated. Gene expression analyses were carried out to determine genes differentially regulated by VASH2. Putative transcription factors that are downstream mediators of gene expression regulated by VASH2 were queried bioinformatically. Dual-luciferase reporter assays and ChIP assays were performed to confirm transactivation of target genes following VASH2 overexpression or knockdown.

**Results:**

VASH2 protein expression was higher in human pancreatic cancer than in paired adjacent tissues and elevated VASH2 levels were associated with gemcitabine chemoresistance. In cell line models of pancreatic cancer, VASH2 expression induced gemcitabine chemoresistance in vitro and in vivo. It was discovered that expression of ribonucleotide reductase regulatory subunit M2 (RRM2) is regulated by VASH2; immunohistochemical analysis demonstrated a positive association of VASH2 expression and RRM2 expression in human pancreatic cancer tissues. Bioinformatics analyses revealed that induction of the Jun proto-oncogene (JUN) by VASH2 is responsible for upregulation of RRM2 expression; this JUN-dependent regulation of RRM2 by VASH2 was confirmed by chromatin immunoprecipitation and dual luciferase reporter assays, which demonstrated that JUN directly binds with the RRM2 promoter to activate transcription.

**Conclusions:**

These data suggest that VASH2 reduces the chemosensitivity to gemcitabine in pancreatic cancer cells via JUN-dependent transactivation of RRM2.

**Electronic supplementary material:**

The online version of this article (doi:10.1186/s12943-017-0619-6) contains supplementary material, which is available to authorized users.

## Background

Pancreatic cancer carries a uniformly poor prognosis with low surgical resection rate and short survival time, and improvement in prognosis, even for resectable cases, is a persistent clinical challenge. Gemcitabine is the first-line chemotherapy drug for adjuvant treatment of pancreatic cancer, but has demonstrated limited ability to improve the prognosis of patients with pancreatic cancer. The poor efficacy of gemcitabine in pancreatic cancer is due to chemoresistance of the cancer cells. The mechanism of chemoresistance to gemcitabine is elusive, and it is necessary to define gemcitabine-resistance mechanisms in pancreatic cancer to identify novel targets and develop means to overcome chemoresistance to gemcitabine.

The Vasohibin family contains two members: Vasohibin 1 (VASH1) and Vasohibin 2 (VASH2) [1]. VASH1 is located in the cytoplasm of endothelial cells and was first identified as a negative regulator of angiogenesis [2, 3]. VASH2 is a homolog of VASH1, and was shown to stimulate angiogenesis in a mouse model of hypoxia-induced subcutaneous angiogenesis [3]. We have demonstrated that VASH2 protein expression can be detected in both the nuclear and cytoplasmic compartments [4]. Recently, VASH2 has been demonstrated to be involved in the malignant behavior of a number of malignancies, including hepatic cancer [5, 6], ovarian cancer [7, 8], endometrial cancer [9], gastrointestinal cancers [10], breast cancer [11], and pancreatic cancer [12]. Kim JC et al. have reported that VASH2 promotes tumor progression and is associated with a poor clinical outcome in pancreatic ductal adenocarcinoma [12]. However, the relation between VASH2 expression and efficacy of chemotherapy remains to be elucidated.

We previously produced rabbit polyclonal anti-human VASH2 antibodies which were successfully applied in immunoblotting and immunohistochemical analyses of human liver cancer and adjacent normal tissues [4, 13], breast cancer [11], and multiple other human cancer and normal tissues [14]. Here, we investigated the expression of VASH2 in human pancreatic cancer and analyzed the relationship between VASH2 expression and clinical features. We also investigated the function and mechanism of VASH2 in human pancreatic cancer using in vitro and in vivo models. We demonstrate that VASH2 is overexpressed in human pancreatic cancer and functions as a gemcitabine-resistance factor by Jun proto-oncogene (JUN) dependent transactivation of ribonucleotide reductase regulatory subunit M2 (RRM2).

## Methods

### Clinical samples

Human pancreatic cancer tissue (pancreatic ductal adenocarcinoma) and paired adjacent normal pancreas tissue were obtained from 102 patients who underwent surgical resection at Jiangsu Province Hospital during January 2012 to December 2013. All patients were treatment-naive to chemotherapy and radiotherapy prior to surgery. This study was approved by the Ethics Committee of the First Affiliated Hospital with Nanjing Medical University. All surgical specimens were obtained after explanation to the patient and after his/her written and signed informed consent. A portion of the pancreatic cancer patients (30/102) received adjuvant chemotherapy: gemcitabine was administered on days 1, 8, and 15 for four to six cycles (four weeks per cycle) post-operation. The remaining portion of pancreatic cancer patients (72/102) received no adjuvant chemotherapy or radiotherapy. Patients who were alive at last follow-up were censored for survival analysis.

### Animals

Five-week-old male nude mice (BALB/cA-nu [nu/nu]) were obtained from Vital River Laboratories (Beijing, China). All experimental procedures were approved by the Animal Care and Use Subcommittee of Nanjing Medical University.

### Cell culture

SW1990 and PANC-1 human pancreatic cancer cells were obtained from the Shanghai Cell Bank (Type Culture Collection Committee, Chinese Academy of Sciences). Cells were maintained in DMEM (Gibco, Thermo Fisher Scientific, USA) containing 10% FBS (Gibco). All cells were cultures in a humidified incubator at 37°C and 5% CO_2_.

### Establishment of stable cell lines using plasmid and lentivirus

Lentiviral constructs were designed for the overexpression or knockdown VASH2, as previously described [5]. PANC-1 cells were stably transfected with Lv-CMV-VASH2 to overexpress VASH2 (PANC-1-VASH2); PANC-1 cells were stably transfected with the control plasmid Lv-CMV-EGFP (PANC-1-EGFP); SW1990 cells were stably transfected with VASH2-targeting lentiviral shRNA for stable knockdown of VASH2 (SW1990-shVASH2); SW1990 cells were stably transfected with scrambled lentiviral shRNA (SW1990-scramble). JUN expressing plasmid was obtained from GeneCopoeia (EX-B0091, Guangzhou, China).

### Quantitative RT–PCR

Total RNA was extracted using RNAiso plus reagent and cDNA was prepared using the Primescript RT Reagent (TAKARA, Dalian, China). Quantitative RT–PCR was performed using the ABI Step One Plus Real-Time-PCR System (Applied Biosystems, USA) with SYBR Green Master Mix (Applied Biosystems). RT-PCR was performed for RRM2, JUN, and GAPDH. GAPDH expression was used as a reference to determine fold changes for the target genes using the comparative Ct method [5]. The sequences for primers against RRM2, JUN, and GAPDH are provided in Additional file 1.

### Immunoblotting

Whole cell lysates were prepared in radioimmunoprecipitation assay buffer (Beyotime, Nantong, China) and blotted using the following primary antibodies: rabbit polyclonal anti-VASH2 (prepared as described in [4]); rabbit polyclonal anti-RRM2 (cat. no. ab57653, Abcam, USA); rabbit polyclonal anti-JUN (cat. no. sc-1694, Santa Cruz, USA). The secondary antibodies used for detection were horseradish peroxidase-conjugated goat anti-mouse IgG and horseradish peroxidase-conjugated donkey anti-rabbit IgG (both CWBIO, Beijing, China).

### Immunohistochemistry

Immunohistochemical staining of the clinical samples was performed as previously described [4]. Primary antibodies: rabbit polyclonal anti-VASH2 [4]; rabbit polyclonal anti-RRM2 (cat. no. ab57653, Abcam). VASH2 and RRM2 staining intensity were semi-quantitatively scored by two pathologists as follows: negative: 0; weak staining: 1; moderate staining: 2; and strong staining: 3. Unless otherwise specified (as in the cancer vs. adjacent normal tissue analysis), degree of VASH2 staining refers to staining within pancreatic cancer cells.

### Analysis of cellular apoptosis

Using Annexin V-PE/7-AAD Apoptosis Detection Kit (Becton Dickinson, San Jose, CA, USA), cellular apoptosis was assessed by flow cytometry (Becton Dickinson). Cells were cultured with gemcitabine (25nM or 50nM) for 48 h [15]. Cells were collected, washed with PBS, and suspended in 100 μL binding buffer, stained with 5μL of Phycoerythrin (PE)–Annexin-V and 5 μL of 7-AAD for 15 min in the dark. The stained cells were analyzed immediately.

### In vivo tumorigenesis

5 × 10^6^ of PANC-1-EGFP or PANC-1-VASH2 were bilaterally subcutaneously injected into the flanks of nude mice; as control, 1 × 10^6^ SW1990-scramble or SW1990-shVASH2 cells were bilaterally subcutaneously injected into the flanks of mice. Once tumor size reached 0.5-1.0cm, mice were euthanized and the xenograft tumors were harvested, cut into small pieces (1mm^3^), and then subcutaneously re-implanted into nude mice. This process was performed twice. Finally, PANC-1-EGFP/PANC-1-VASH2 (*n* = 12) or SW1990-scramble/SW1990-shVASH2 (*n* = 14) xenograft tumor pieces were subcutaneously implanted in the back of the same mice in symmetrical positions on both sides. Mice were divided into four groups: PANC-1 group (*n* = 6): mice implanted with PANC-1-EGFP/PANC-1-VASH2 tumor pieces *without* gemcitabine chemotherapy; PANC-1-GZ group (*n* = 6): mice implanted with PANC-1-EGFP/PANC-1-VASH2 tumor pieces *with* gemcitabine chemotherapy; SW1990 group (*n* = 7): mice implanted with SW1990-scramble/SW1990-shVASH2 tumor pieces *without* gemcitabine chemotherapy; SW1990-GZ group (*n* = 7): mice implanted with SW1990-scramble/SW1990-shVASH2 tumor pieces *with* gemcitabine chemotherapy. Administration of chemotherapy began when the tumor diameter reached 3-5mm: every Tuesday and Saturday gemcitabine was injected intraperitoneally at 100mg/kg; the SW1990-GZ group was treated for 3 weeks; the PANC-1-GZ group was treated for four consecutive weeks. Tumors were weighed by electronic scales. Tumor control rate was calculated as the following formula:

$$ \mathrm{Tumor}\ \mathrm{control}\ \mathrm{rate} = \left(\mathrm{control}\ \mathrm{group}\ \mathrm{tumor}\ \mathrm{weight}\ \hbox{--}\ \mathrm{VASH}2\ \mathrm{overexpressing}/\mathrm{knockdown}\ \mathrm{group}\ \mathrm{tumor}\ \mathrm{weight}\right) \times 100/\mathrm{control}\ \mathrm{group}\ \mathrm{tumor}\ \mathrm{weight}. $$

A higher tumor control rate indicates that the tumor size is smaller in experimental compared to control group, and a lower tumor control rate indicates that tumor size is greater in experimental group compared to control.

### TdT-Mediated dUTP-Biotin Nick End-Labeling (TUNEL)

Xenograft tumor tissues were embedded in paraffin and sectioned for the TUNEL assay. TUNEL staining was performed by Biohelper Nanjing company (Biohelper, Nanjing, China) using an *in situ* cell death detection kit (Roche, Switzerland) according to the manufacturer's instructions. TUNEL assay results were determined by counting 1,000 cells in six randomly selected fields per sample.

### Gene expression array

Samples of PANC-1-EGFP or PANC-1-VASH2 cells were prepared for gene expression analysis using NimbleGen 12x135K microarrays (Roche Applied Science, Switzerland). Arrays were scanned using an Axon GenePix 4000B microarray scanner (Molecular Devices, CA, USA). Scanned images were imported into NimbleScan software (version 2.6, Roche Applied Science, Switzerland) for gene expression data analysis. Differentially expressed genes were identified through Fold Change filtering. Genes with fold changes ≥ 3 or ≤ 0.33 were selected for further analysis.

### siRNA

Three small interfering RNAs (GenePharma, Shanghai, China) were used for JUN knockdown; siRNA sequence information is provided in Additional file 1. Lipofectamine RNAiMAX transfection reagent (Invitrogen, Thermo Fisher Scientific, USA) was used for siRNA transfection.

### Chromatin immunoprecipitation (ChIP)

ChIP was performed using the Magna ChIP Chromatin Immuno Precipitation kit (Millipore, Billerica, MA, USA). Immunoprecipitations were carried out with anti-c-Jun (H79) (cat. no. sc1694, Santa Cruz) antibody. Precipitated DNA was purified and used as a template for PCR reactions. Primers used for PCR in chromatin immunoprecipitation experiments are described in Additional file 1.

### Dual luciferase reporter assay

The *RRM2* promoter (-2147/+1 relative to the transcription start site) [16] containing a JUN binding site (-643/-630 relative to the transcription start site) was synthesized (GenScript, Nanjing, China) and ligated into pGL3 basic reporter vector (Promega, Madison, WI, USA) to create PGL3-WT. A reporter vector containing a mutated JUN binding site in the *RRM2* promoter was constructed (PGL3-MUT; TTTACATGAGTCAT → GCGCAGGACACAGC). Reporter plasmids were co-transfected with a Renilla luciferase expression plasmid (pRL-TK; Promega) as transfection control. Cells were cultured for 24 h following transfection, and luciferase activity was measured using the Dual Luciferase Reporter Assay System (Promega). The relative promoter activity was calculated as firefly luminescence/Renilla luminescence.

### Statistical analysis

Statistical analysis was performed using SPSS 13.0 (SPSS, Chicago, IL, USA) and GraphPad Prism 5.01 (GraphPad Software Inc., San Diego, CA, USA). Data were shown as mean ± S.E.M. The experimental and control groups were compared using the Student’s *t-*test. The Pearson’s Chi-square test was used to compare differences in proportions of VASH2 staining intensity. Kaplan-Maier survival analysis was used to compare survival times. Spearman correlation coefficients were calculated to compare the expression of VASH2 and RRM2. Significant differences are indicated with * (*P* < 0.05).

## Results

### Correlation between VASH2 expression and histopathologic features of pancreatic cancer

We investigated the levels of VASH2 expressed in 102 human pancreatic cancer tissue samples and paired adjacent tissues by immunohistochemical analysis. General clinical information of the patients is shown in Table 1. Correlations between VASH2 staining intensity and histopathologic features of the 102 pancreatic cancer cases are shown in Table 2. Representation of immunostaining pictures for weak and strong VASH2 staining in pancreatic cancer tissues were shown in Additional file 2. The proportion of specimens exhibiting middle/strong staining for VASH2 was significantly higher in pancreatic cancer tissue samples (56.9%, 58/102) than in adjacent normal tissue samples (24.5%, 25/102; *P* = 0.001). The proportion of specimens with middle/strong staining for VASH2 expression was generally higher in grade 3 pancreatic cancers than in grade 1-2 pancreatic cancers (37/54 [68.5%] vs. 21/48 [43.8%], respectively; *P* = 0.012). The proportion of specimens with middle/strong VASH2 staining was significantly lower in vessel cancerous embolus negative pancreatic cancer tissues than in vessel cancerous embolus positive pancreatic cancer tissues (47/89 [52.8%] vs. 11/13 [84.6%], respectively; *P* = 0.031). Degree of VASH2 staining was not found to associate with pathologic stage, node status, or nerve status of pancreatic cancer samples.

**Table 1:** General information of pancreatic cancer patients (102 cases)

Category	*n* (%)
Gender	
Male	40 (39.2)
Female	62 (60.8)
Age (years)	
Average	61.5 ± 10.9
Range	32-82
Chemotherapy (gemcitabine)	
Yes	30(29.4)
No	72(70.6)
Pathology staging(AJCC 7_th_)	
I	10 (9.8)
II	80 (78.5)
III	12 (10.8)

**Table 2:** Correlation between VASH2 and histopathologic features in 102 cases of pancreatic cancer

Category	*n* (%)	VASH2 staining (%)		*P* value^1^
		Negative/weak	Middle/strong	
Adjacent tissues	102	77(75.5)	25(24.5)	0.001
Cancer tissue	102	44(43.1)	58(56.9)	
Tumor Grade				
G1-G2	48 (47.1)	27(56.2)	21(43.8)	0.012
G3	54 (52.9)	17(31.5)	37(68.5)	
Pathologic stage				
T1-2	15(14.7)	5(33.3)	10(66.7)	0.406
T3-4	87(85.3)	39(44.8)	48(55.2)	
Node status				
Negative	57(55.9)	20(35.1)	37(64.9)	0.065
Positive	45(44.1)	24(53.3)	21(46.7)	
Nerve status				
Negative	34(33.3)	16(47.1)	18(52.9)	0.572
Positive	68(66.7)	28(41.2)	40(58.8)	
Vessel cancerous embolus				
Negative	89(87.3)	42(47.2)	47(52.8)	0.031
Positive	13(12.7)	2(15.4)	11(84.6)	

^1^Pearson’s chi-square test *p*-value

### VASH2 expression is associated with gemcitabine resistance in pancreatic cancer

**Fig. 1:**
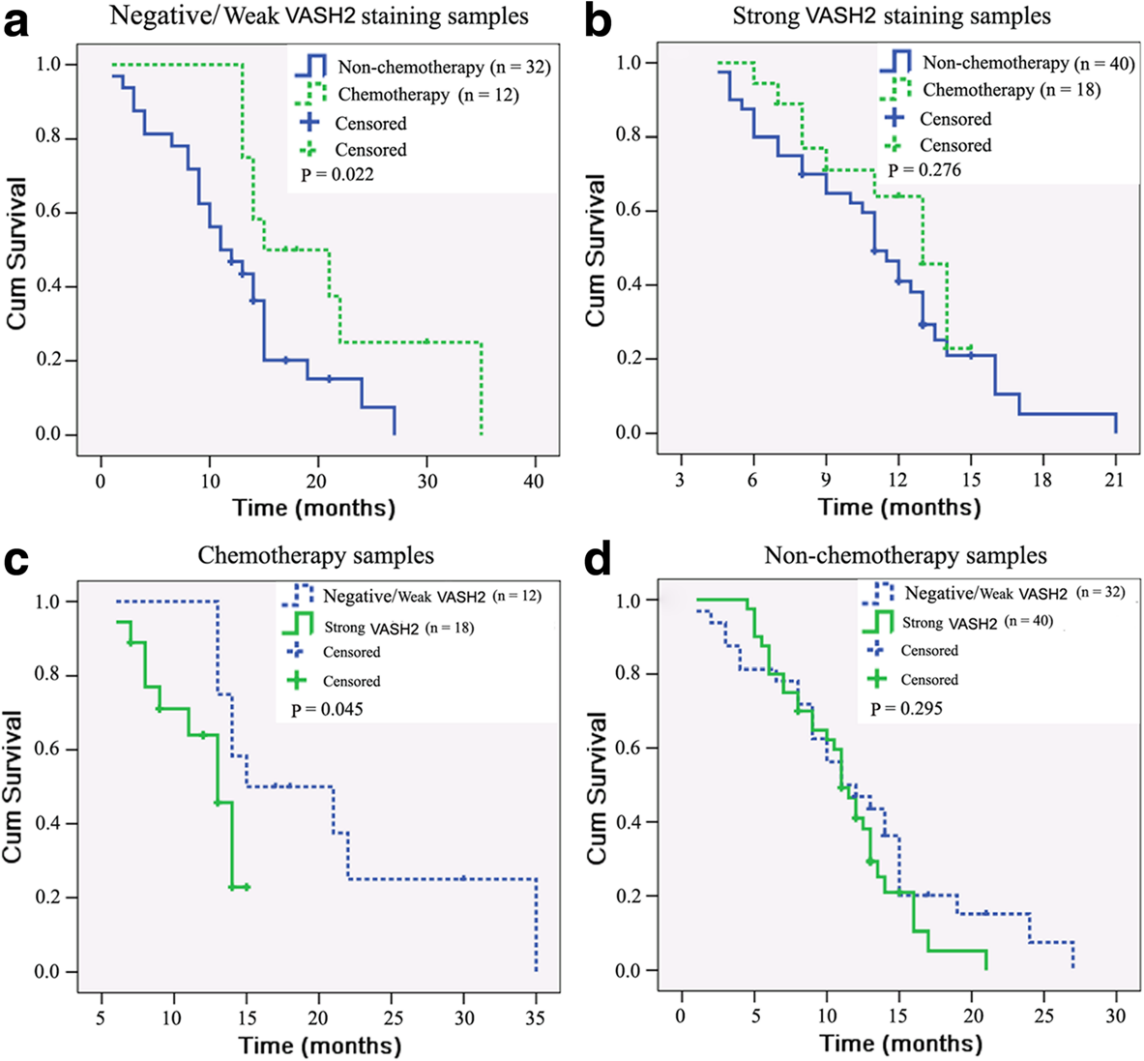
VASH2 expression is associated with gemcitabine resistance in pancreatic cancer. Kaplan-Meier survival analysis results of 102 cases of pancreatic cancer. **a** survival analysis of patients with negative/weak VASH2 staining tumors treated with or without gemcitabine chemotherapy. **b** survival analysis of patients with middle/strong VASH2 staining tumors treated with or without gemcitabine chemotherapy. **c** survival analysis of patients treated with gemcitabine chemotherapy with negative/weak or middle/strong VASH2 staining tumors. **d** survival analysis of patients who received no chemotherapy with negative/weak or middle/strong VASH2 staining tumors

In cases with negative/weak VASH2 staining, patients treated with gemcitabine chemotherapy exhibited significantly better survival than patients not treated with chemotherapy (20.96 ± 2.87 months vs. 12.45 ± 1.34 months, respectively; *P* = 0.022; Fig. 1a). In contrast, among cases with middle/strong VASH2 staining, there was no significant difference in survival time between patients treated with gemcitabine chemotherapy and those not treated with chemotherapy (11.99 ± 0.74 months vs. 11.26 ± 0.73 months, respectively; *P* = 0.276; Fig. 1b). Among patients treated with gemcitabine chemotherapy, cases with middle/strong VASH2 staining exhibited significantly poorer survival than cases with negative/weak VASH2 staining (Fig. 1c, *P* = 0.045). There was no significant difference in survival time associated with degree of VASH2 staining in patients who were not treated with chemotherapy (Fig. 1d, *P* = 0.295). These results indicate that VASH2 expression may be associated with gemcitabine resistance in pancreatic cancer.

### VASH2 decreased the sensitivity of pancreatic cancer cells to gemcitabine in vitro

To further assess the effect of VASH2 on the sensitivity of pancreatic cancer cells to chemotherapy, we generated human pancreatic cancer cells overexpressing VASH2 (PANC-1-VASH2) and in which VASH2 had been knocked down using a small hairpin RNA (SW1990-shVASH2). Immunoblotting confirmed that the expression of VASH2 in PANC-1-VASH2 cells exceeded that of parental PANC-1 cells transduced to express EGFP; SW1990-shVASH2 cells expressed lower levels of VASH2 than the parental SW1990 cells transfected with a scrambled shRNA (Additional file 3).

**Fig. 2:**
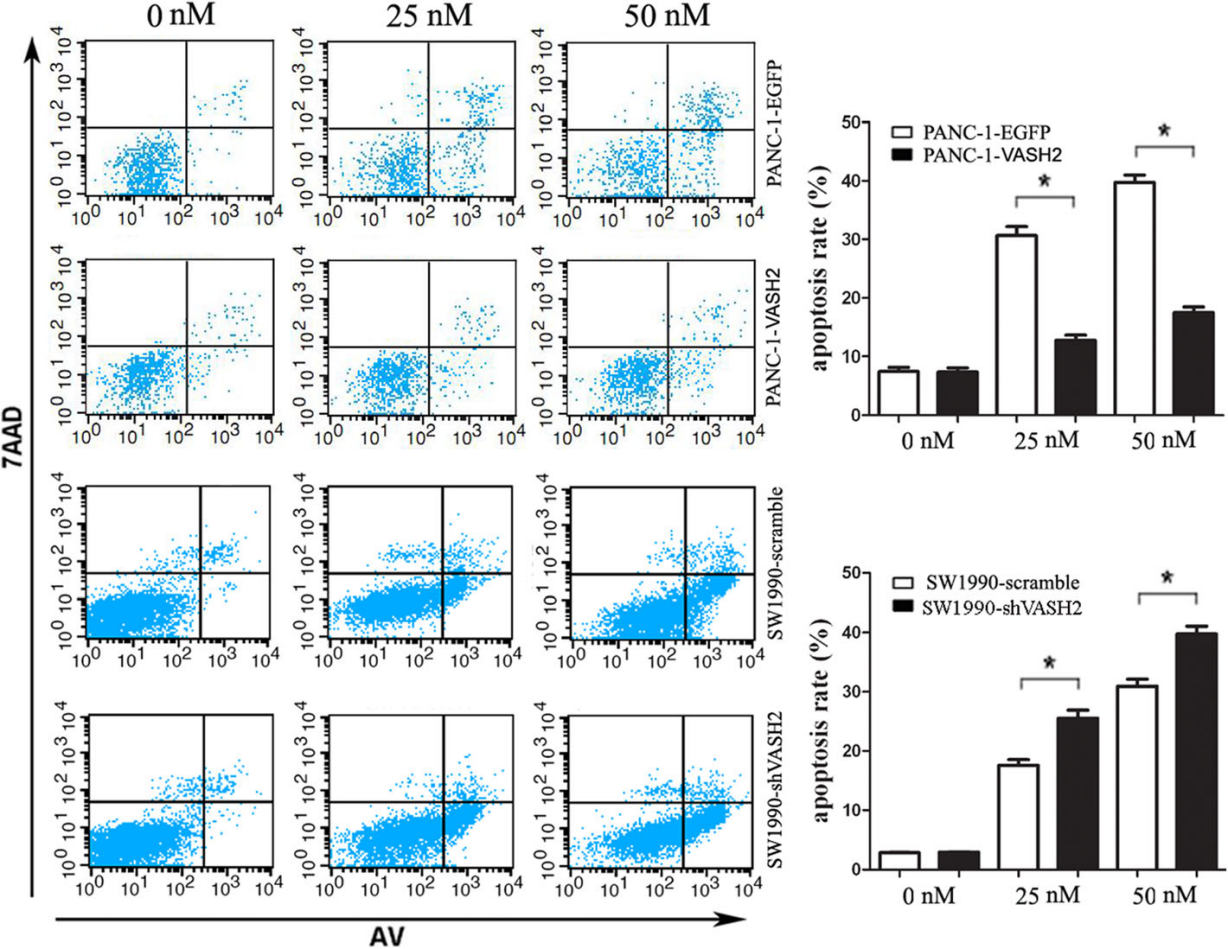
VASH2 decreases the sensitivity of pancreatic cancer cells to gemcitabine in vitro. After treatment with 25 or 50 nM gemcitabine for 48 h, the apoptosis rate of PANC-1-EGFP, PANC-1-VASH2, SW1990-scramble, and SW1990-shVASH2 was indicated by flow cytometric determination of Annexin V and 7-AAD staining. Upper-right + lower-right quadrants indicate apoptotic cells. Apoptosis is quantified in the bar graphs for PANC-1-EGFP and PANC-1-VASH2 (top) and SW1990-scramble and SW1990-shVASH2 (bottom). *P < 0.05

To determine the impact of these changes in VASH2 expression on gemcitabine sensitivity, cellular apoptosis was analyzed in these models after treatment with 25 nM or 50 nM gemcitabine for 48 h. VASH2 overexpression significantly reduced sensitivity to gemcitabine, as PANC-1-VASH2 cells exhibited significantly decreased apoptosis compared to PANC-1-EGFP cells after treatment with 25 nM gemcitabine (12.69% ± 1.62% vs. 30.63% ± 2.65%, respectively; *P* < 0.05; Fig. 2) and 50 nM gemcitabine (17.47% ± 1.67% vs. 39.71% ± 2.21%, respectively; *P* < 0.05; Fig. 2). VASH2 knockdown significantly increased sensitivity to gemcitabine, as SW1990-shVASH2 cells exhibited significantly higher rates of apoptosis than SW1990-scramble cells after treatment with 25 nM gemcitabine (25.52% ± 2.37% vs. 17.59% ± 1.63%, respectively; *P* < 0.05; Fig. 2) and 50 nM gemcitabine (39.77% ± 2.22% vs. 30.93% ± 2.09%, respectively; *P* < 0.05; Fig. 2). Without gemcitabine, the apoptosis rate of VASH2 overexpression/knockdown cells showed no significant difference compared with control groups (Fig. 2, *P* > 0.05). These results indicate that VASH2 decreased the gemcitabine sensitivity of pancreatic cancer cells in vitro.

### VASH2 decreased the sensitivity of pancreatic cancer cells to gemcitabine in vivo

**Fig. 3:**
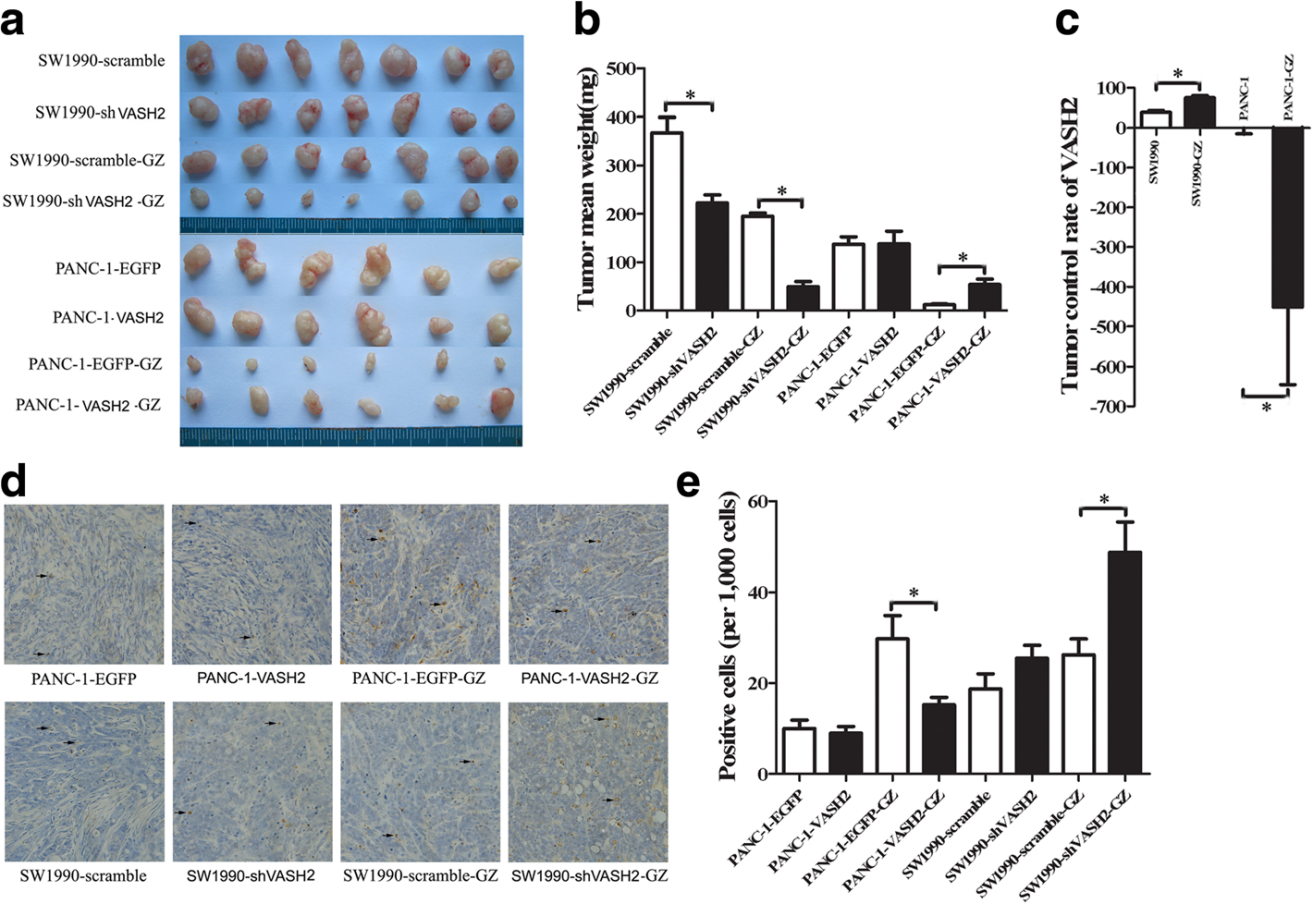
Tumor control rate analysis using subcutaneous tumorigenesis in nude mice. Mice were divided into four groups: PANC-1 group (*n* = 6), mice implanted with PANC-1-EGFP/PANC-1-VASH2 tumor pieces without gemcitabine chemotherapy; PANC-1-GZ group (*n* = 6), mice implanted with PANC-1-EGFP/PANC-1-VASH2 tumor pieces with gemcitabine chemotherapy; SW1990 group (*n* = 7), mice implanted with SW1990-scramble/SW1990-shVASH2 without gemcitabine chemotherapy; SW1990-GZ group (*n* = 7), mice implanted with SW1990-scramble/SW1990-shVASH2 with gemcitabine chemotherapy. **a** resected xenograft tumors from all groups. **b** tumor mean weight analysis. **c** the tumor control rate of VASH2. **d** and **e** representative images of and quantitation of TUNEL staining for apoptosis in the xenograft tumors, as determined by counting 1,000 cells in 6 randomly selected fields. The black arrows show the brown stained TUNEL-positive cells. *P < 0.05

To further investigate the impact of VASH2 on pancreatic cancer sensitivity to gemcitabine in vivo we analyzed the tumor control rate using a xenograft model of subcutaneous tumor growth in nude mice. Images of whole excised tumor masses are shown in Fig. 3a. Untreated SW1990-shVASH2 tumors were smaller than SW1990-scramble tumors (222.7 ± 44.6 mg vs. 367.6 ± 84.4 mg, respectively; *P* < 0.05; Fig. 3b). When treated with gemcitabine, SW1990-shVASH2 tumors were smaller than SW1990-scramble tumors (49.3 ± 28.9 mg vs. 195.0 ± 18.6 mg, respectively; *P* < 0.05, Fig. 3b). Untreated PANC-1-VASH2 xenograft tumors had no significantly different weight than PANC-1-EGFP tumors (137.2 ± 37.7 mg vs. 138.2 ± 64.0 mg, respectively; *P* > 0.05, Fig. 3b). However, when treated with gemcitabine, PANC-1-VASH2 tumors were significantly larger than PANC-1-EGFP tumors (54.0 ± 27.2 mg vs. 12.2 ± 4.4 mg, respectively; *P* < 0.05, Fig. 3b). To analyze the relationship between VASH2 perturbation and gemcitabine chemosensitivity, the tumor control rate was calculated (the intervention factor was VASH2 overexpression or knockdown). In SW1990 group, the tumor control rate was significantly lower than SW1990-GZ group (Fig. 3c, *P* < 0.05), and the tumor control rate of PANC-1 group was significantly higher than PANC-1-GZ group (Fig. 3c, *P* < 0.05). These results suggest that VASH2 knockdown in pancreatic cancer cells increases the growth inhibitory effect of gemcitabine chemotherapy, and overexpression of VASH2 reduces the growth inhibitory effect of gemcitabine chemotherapy.

TUNEL analysis was employed to determine the impact of VASH2 modulation on apoptosis of pancreatic cancer cells in vivo (representative images of TUNEL assay from xenograft tumors shown in Fig. 3d). Following treatment with gemcitabine, SW1990-shVASH2 with VASH2 knockdown exhibited increased cellular apoptosis compared to SW1990-scramble cells (48.8 ± 13.4 vs. 26.3 ± 6.7; *P* < 0.05; Fig. 3e). After treatment with gemcitabine, PANC-1-VASH2 cells overexpressing VASH2 demonstrated significantly decreased apoptosis than PANC-1-EGFP cells (15.3 ± 3.3 vs. 29.8 ± 10.3; *P* < 0.05; Fig. 3e). Without gemcitabine, there were no significant differences in apoptosis rate between VASH2 overexpression/knockdown cells and control cell lines (Fig. 3e). These data indicate that VASH2 can decrease the sensitivity of pancreatic cancer cells to gemcitabine in vivo.

### VASH2 regulates the expression of RRM2

**Fig. 4:**
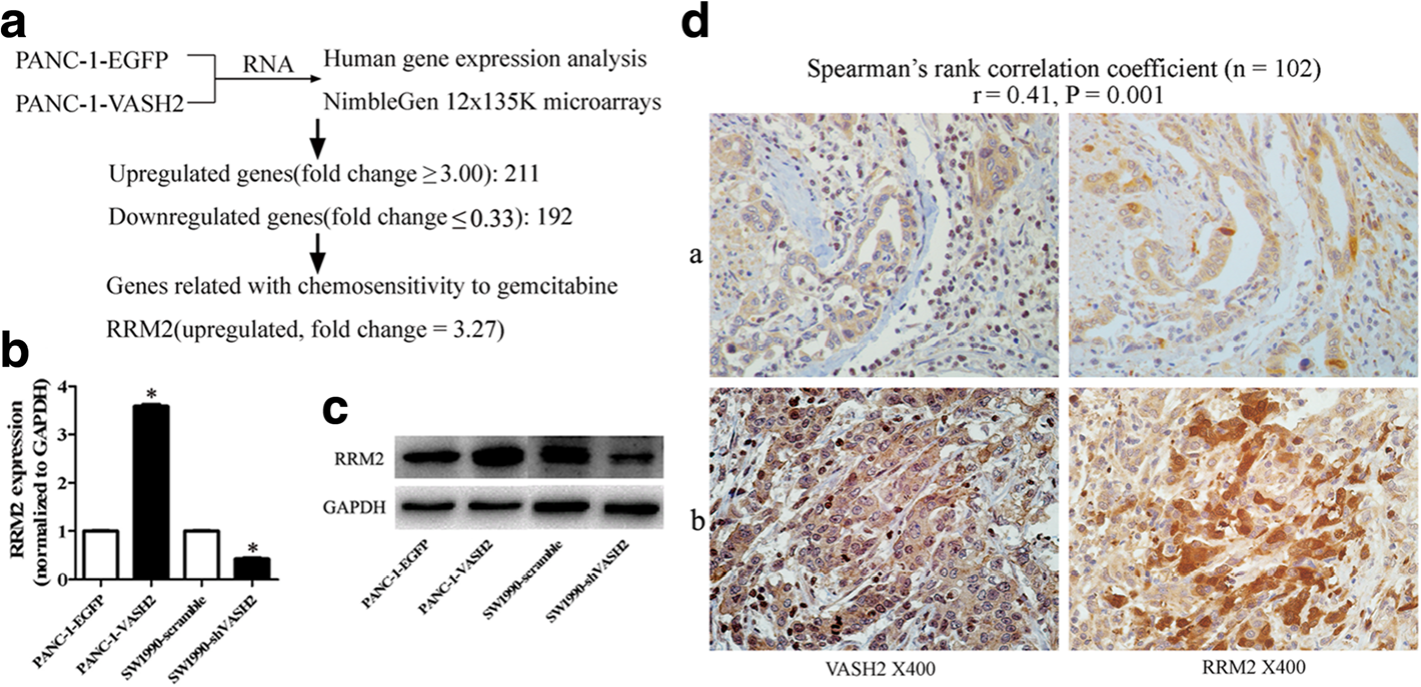
VASH2 regulates the expression of RRM2 and VASH2 expression correlates with RRM2 expression in human pancreatic cancer. **a** human gene expression NimbleGen 12x135K microarrays within PANC-1-EGFP/PANC-1-VASH2 cells. Fold changes ≥ 3 (upregulation; 211 genes) or ≤ 0.33 (downregulation, 192 genes) were selected as significantly altered genes for subsequent analysis. RRM2 (a typical gemcitabine resistance associated gene) was upregulated by VASH2 (fold change = 3.27). **b** and **c** qPCR analysis and immunobloting test of RRM2 in PANC-1-VASH2, PANC-1-EGFP cells, SW1990-shVASH2 and SW1990-scramble cells. **d** representative images of RRM2 and VASH2 staining in human pancreatic cancer tissues expressing low (*a*) and high (*b*) levels of VASH2 are shown. (Spearman’s rank correlation coefficient, r = 0.41, *P* = 0.001). *P < 0.05

Using the NimbleGen 12x135K microarrays, gene expression analysis were performed in PANC-1-EGFP/PANC-1-VASH2 cells and differentially expressed genes were identified through Fold Change filtering. Filtering based on a threshold fold changes of ≥ 3 for upregulated genes or ≤ 0.33 for downregulated genes identified 211 significantly upregulated genes and 192 significantly downregulated genes (Additional file 4). *RRM2*, a typical gemcitabine resistance associated gene was upregulated by VASH2 (fold change = 3.27; Fig. 4a). RRM2 mRNA and protein levels were detected in PANC-1-EGFP, PANC-1-VASH2, SW1990-scramble, and SW1990-shVASH2 cells. *RRM2* expression was significantly higher in PANC-1-VASH2 cells than in PANC-1-EGFP, while *RRM2* expression in SW1990-shVASH2 cells was significantly lower than in SW1990-scramble cells (Fig. 4b). Immunoblot analysis confirmed that RRM2 protein was expressed at higher levels in PANC-1-VASH2 cells than PANC-1-EGFP cells, and at lower levels in SW1990-shVASH2 cells than SW1990-scramble cells (Fig. 4c). These data suggest that the gemcitabine metabolism associated gene *RRM2* is regulated by VASH2.

### Expression of VASH2 correlates positively with RRM2 expression in human pancreatic cancer

Human pancreatic cancer tissues from 102 patients were subjected to immunohistochemical staining for VASH2 and RRM2. Two representative examples are presented in Fig. 4d. Typically, weak RRM2 immunoreactivity was observed in specimens that were weakly positive for VASH2, whereas strong RRM2 immunoreactivity was detected in specimens that were strongly positive for VASH2. In the 102 specimens, VASH2 expression was significantly positively correlated with RRM2 expression (Spearman’s rank correlation coefficient = 0.41, *P* = 0.001).

### JUN is induced by VASH2 and binds to the promoter of RRM2, increases transactivation of RRM2

The promoter region of *RRM2* was analyzed by SABiosciences (http://www.sabiosciences.com/chipqpcrsearch.php) to determine the factor(s) reposnisble for transactivation of of *RRM2*. Six potential transcription factors were identified: Heat Shock Transcription Factor 2, Nuclear Factor Kappa B Subunit 1, E2F Transcription Factor 1, Sp1 Transcription Factor, CAMP Responsive Element Binding Protein, and JUN. Of these, JUN was the only transcription factor predicted to bind the *RRM2* promoter that was among the differentially expressed genes induced by VASH2 as determined from the NimbleGen 12x135K microarrays (fold change = 3.94).

**Fig. 5:**
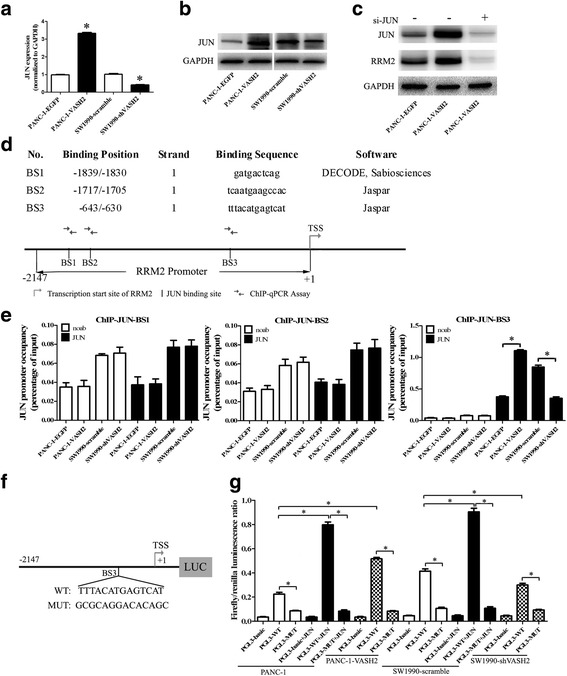
JUN is upregulated by VASH2, binds to the RRM2 promoter, and increases transactivation of RRM2. **a** and **b** qPCR analysis and immunobloting for JUN in PANC-1-VASH2, PANC-1-EGFP, SW1990-shVASH2, and SW1990-scramble cells. **c** JUN and RRM2 immunobloting in PANC-1-EGFP and PANC-1-VASH2 transfected with siRNA against JUN. **d** diagram of three predicted JUN-binding sites within RRM2 promoter (-2147/+1) by SABiosciences and Jaspar online software. **e** ChIP (JUN) analysis for three predicted JUN-binding sites of RRM2 promoter in PANC-1-EGFP, PANC-1-VASH2, SW1990-scramble and SW1990-shVASH2 cells. **f** and **g** dual luciferase reporter assays for PGL3-WT/PGL3-MUT reporter activity co-transfected with or without a JUN expression plasmid in PANC-1, PANC-1-VASH2, SW1990-scramble, and SW1990-shVASH2 cells. The relative promoter activity was calculated as firefly luminescence/Renilla luminescence. *P < 0.05

JUN protein and mRNA expression was detected by western blotting and quantitative RT-PCR analyses, respectively, in PANC-1-EGFP, PANC-1-VASH2, SW1990-scramble, and SW1990-shVASH2 cells. JUN mRNA and protein expression was significantly upregulated by overexpression of VASH2 and downregulated by knockdown of VASH2 expression in the respective cell line models (Fig. 5a, b). Furthermore, transfection with siRNA against JUN mRNA resulted in a strong downregulation of JUN and RRM2 protein in PANC-1-VASH2 cells (Fig. 5c; siRNA knockdown of JUN mRNA demonstrated in Additional file 5) siRNA. These results suggest that the expression regulation of RRM2 by VASH2 was JUN dependent.

Three JUN-binding sites were identified within RRM2 promoter using SABiosciences (http://www.sabiosciences.com/chipqpcrsearch.php) and Jaspar software (http://jaspar.genereg.net/cgi-bin/jaspar_db.pl): (-2147/+1): BS1, -1839/-1830; BS2, -1717/-1705; BS3, -643/-630 (Fig. 5d). ChIP analysis demonstrated that JUN was recruited to the BS3 locus but not BS1 or BS2 on the RRM2 promoter (Fig. 5e). JUN protein was recruited to the BS3 locus in the RRM2 promoter more efficiently in PANC-1-VASH2 cells than in PANC-1-EGFP cells, while less efficiently in SW1990-scramble than SW1990-shVASH2 (Fig. 5e).

Using dual luciferase reporter assays, we investigated whether JUN directly activates transcription of RRM2. RRM2 promoter luciferase reporter plasmids (PGL3-WT/PGL3-MUT) were co-transfected with a Renilla luciferase expression plasmid (pRL-TK) with or without a JUN expression plasmid in PANC-1, PANC-1-VASH2, SW1990-scramble, and SW1990-shVASH2 cells (Fig. 5f). The relative promoter activity was calculated as firefly luminescence/Renilla luminescence. The mutant RRM2 promoter (PGL3-MUT) has strongly reduced reporter activity compared to the wild-type RRM2 promoter (PGL3-WT). Overexpression of JUN from a JUN expression plasmid increased reporter activity from the wild-type RRM2 promoter but not the mutant RRM2 promoter (Fig. 5g). Reporter activity from the wild-type RRM2 promoter but not from the mutant RRM2 promoter was significantly increased in PANC-1-VASH2 cells compared with PANC-1 cells, and significantly decreased in SW1990-shVASH2 cells compared with SW1990-scramble cells (Fig. 5g). The mutant RRM2 promoter failed to elicit a response to either elevated endogenous levels of JUN or to forced overexpression of JUN from the JUN expression plasmid. These data indicate that VASH2 upregulates JUN and JUN activates RRM2 transcription through direct binding to the RRM2 promoter, establishing a JUN-dependent transactivation of RRM2 downstream of VASH2.

## Discussion

VASH2 has been implicated in tumor progression [5–12]. In this study, we investigated the expression of VASH2 in human pancreatic cancer, and found significantly higher levels of VASH2 in pancreatic cancer tissues than in paired cancer-adjacent normal tissue. VASH2 expression was associated with higher tumor grade and more vessel cancerous embolus. Survival analysis indicated that tumors that were negative/weak for VASH2 staining were more sensitive to gemcitabine chemotherapy than tumors exhibiting middle/strong VASH2 staining, indicating that VASH2 may be related with gemcitabine sensitivity in pancreatic cancer. To further investigate the involvement of VASH2 in gemcitabine resistance, we created pancreatic cancer models of VASH2 overexpression and knockdown, and observed that VASH2 inhibited gemcitabine-induced apoptosis in vitro and in vivo.

Gemcitabine treatment is one of the main chemotherapeutic approaches for advanced pancreatic cancer. Gemcitabine has been shown to improve survival for patients with pancreatic cancer, although the improvement in survival time remains short, due to high rates of resistance of pancreatic cancer to gemcitabine [17]. Sensitivity or resistance of pancreatic cancer cells to gemcitabine can be regulated by the activity of genes related to gemcitabine metabolism. Gemcitabine is taken up into cells primarily by human concentrative nucleoside transporter 1 and 3 (hCNT1 and hCNT3) and by human equilibrative nucleoside transporter (hENT1) [18]. The expression of these nucleoside transporters is correlated with chemosensitivity and patient survival [19–23]. After being taken into the cells, gemcitabine is activated by deoxycytidine (dCK) [24, 25]. Thus, hENT1, hCNT1, hCNT3, and dCK positively contribute to gemcitabine activity and to cancer cells' sensitivity to gemcitabine. On the other hand, ribonucleotide reductases (RRM1 and RRM2) and multidrug resistance-associated protein channels (MRP3, MRP4 and MRP 5) contribute to gemcitabine resistance [26, 27].

Here we report that the gemcitabine metabolism related gene, RRM2, is upregulated in pancreatic cancer models of VASH2 overexpression. Furthermore, RRM2 expression was decreased in a pancreatic cell line model with VASH2 knockdown. Immunohistochemical analysis demonstrated that the expression of VASH2 was positively related to the RRM2 in human pancreatic cancer tissues. Collectively, these results indicate that VASH2 induces gemcitabine resistance via upregulation of RRM2 in human pancreatic cancer.

We discovered that the JUN transcription factor is induced by VASH2 overexpression. Moreover, JUN is the only transcription factor significantly differentially expressed following perturbation of VASH2 expression that is predicted to bind to the RRM2 promoter. JUN was significantly upregulated in VASH2 overexpressing cells and significantly downregulated in VASH2 knockdown cells. siRNA againt JUN decreased RRM2 protein, which was upregulated by VASH2. The regulation of RRM2 expression by VASH2 was found to be JUN dependent. Using SABiosciences and Jaspar online software, three JUN binding sites were predicted within the RRM2 promoter; ChIP analysis for JUN confirmed the presence of a bonafide JUN-binding site in the RRM2 promoter. Using dual luciferase reporter assays, we confirmed that JUN directly activates the transcription of RRM2. These data indicate that VASH2 can upregulate JUN, leading to JUN-dependent transcriptional activation of RRM2 via direct binding to the RRM2 promoter.

It is intresting that SW1990-shVASH2 and SW1990-scramble have a difference in their tumor weight, but PANC-1-VASH2 and PANC-1-EGFP tumors do not have (Fig. 3). This effect was also found in HepG2 cells and reported by Xue Xiaofeng et al [5]. One possible reason was that in the control of cell proliferation, PANC-1-EGFP already had sufficient VASH2 expression, and the extra VASH2 in PANC-1-VASH2 cells did not promote cell proliferation. This effect was the same in vivo in SW1990-shVASH2, SW1990-scramble, PANC-1-VASH2 and PANC-1-EGFP cells (data not shown).

## Conclusion

RRM2 has recently emerged as an important factor implicated in the resistance to gemcitabine chemotherapy [28–31]. Here, we found that VASH2 is expressed at high levels in human pancreatic cancer cells and acts as a gemcitabine-resistance factor, and the expression of RRM2 could be upregulated by VASH2 in a JUN-dependent manner. Therefore, VASH2 may represent a novel target for anti-chemoresistance therapy in the gemcitabine chemotherapy of pancreatic cancer; VASH2 may also be used as a marker to guide the gemcitabine chemotherapy of pancreatic cancer. However, the precise pathway by which VASH2 regulates JUN needs further investigation.

## Additional files

Additional file 1: Sequences of primers and siRNAs. (DOC 26 kb)
Additional file 2: Representation of immunostaining pictures for weak and strong VASH2 staining in pancreatic cancer tissues. (TIF 3090 kb)
Additional file 3: Expression of VASH2 in stably transfected PANC-1 and SW1990 pancreatic cancer cells. PANC-1 cells were transfected with a vector expressing EGFP (PANC-1-EGFP) or VASH2 (PANC-1-VASH2), and SW1990 cells were transduced with a scrambled shRNA (SW1990-scramble) or a shRNA targeting VASH2 (SW1990-shVASH2). VASH2 protein expression was assessed by immunoblotting. (TIF 294 kb)
Additional file 4: Human gene expression analysis results using NimbleGen 12x135K microarrays. Differentially expressed genes were identified through Fold Change filtering. Fold change cutoffs of ≥ 3 (upregulation, 211 genes) or ≤ 0.33 (downregulation, 192 genes) were used to identify genes that were significantly differentially expressed. (XLS 69 kb)
Additional file 5: mRNA expression of JUN in PANC-1 pancreatic cancer cells transfected with different JUN-targeting siRNA. (TIF 454 kb)

## Abbreviations

ChIP: Chromatin immunoprecipitation
dCK: Deoxycytidine
hCNT1 and hCNT3: Human concentrative nucleoside transporter 1 and 3
hENT1: Human equilibrative nucleoside transporter
JUN: Jun proto-oncogene
MRP3, MRP4 and MRP 5: Multidrug resistance-associated protein channel 3, 4 and 5
RRM1: Ribonucleotide reductases 1
RRM2: Ribonucleotide reductase regulatory subunit M2
S.E.M: Standard error of measurement
TUNEL: TdT-Mediated dUTP-Biotin Nick End-Labeling
VASH1: Vasohibin 2
VASH2: Vasohibin 2
