# Supplementary material for: Vasohibin 2 reduces chemosensitivity to gemcitabine in pancreatic cancer cells via Jun proto-oncogene dependent transactivation of ribonucleotide reductase regulatory subunit M2
Source: Mol Cancer. 2017 Mar 21;16:66. doi: 10.1186/s12943-017-0619-6 (PMC5360034; doi:10.1186/s12943-017-0619-6)
Supplement: Additional file 1: — Sequences of primers and siRNAs. (DOC 26 kb) [file 12943_2017_619_MOESM1_ESM.doc]

**qRT-PCR primer sequences:**

F-h-c-Jun: 5′-TCGACATGGAGTCCCAGGA-3′

R-h-c-Jun: 5′-GGCGATTCTCTCCAGCTTCC-3′

F-h-RRM2: 5′-CCACGGAGCCGAAAACTAAAG-3′

R-h-RRM2: 5′-CTCTGCCTTCTTATACATCTGCC-3′

F-h-GAPDH: 5'-TACTAGCGGTTTTACGGGCG-3’

R-h-GAPDH: 5'-TCGAACAGGAGGAGCAGAGAGCGA-3'

**ChIP primer sequences:**

ChIP-BS1-F: 5'-AACGAGCACCGAGGGAA-3'

ChIP-BS1-R: 5'-GGTGAAGTCGCCATCCTC-3'

ChIP-BS2-F: 5'-CGGCGTCTTCTACAATGG-3'

ChIP-BS2-R: 5'-AACCCTCGTTTCGGTTGC-3'

ChIP-BS3-F: 5'-AGGCAAGGGTGACAATAGGG-3'

ChIP-BS3-R: 5'-GTGGACTGTTAATGCGGTGAG-3'

**siRNA for JUN (sense, 5’→3’):**

c-Jun siRNA_1_: GGCACAGCUUAAACAGAAA-dTdT

c-Jun siRNA_2_: GCGGGAGGCAUCUUAAUUA-dTdT

c-Jun siRNA_3_: CCAAGAACGUGACAGAUGA-dTdT

control siRNA: AGGAGAUAUUUCGAGGCUU-dTdT
