# Supplementary figures and images for: Vasohibin 2 reduces chemosensitivity to gemcitabine in pancreatic cancer cells via Jun proto-oncogene dependent transactivation of ribonucleotide reductase regulatory subunit M2
Source: Mol Cancer. 2017 Mar 21;16:66. doi: 10.1186/s12943-017-0619-6 (PMC5360034; doi:10.1186/s12943-017-0619-6)

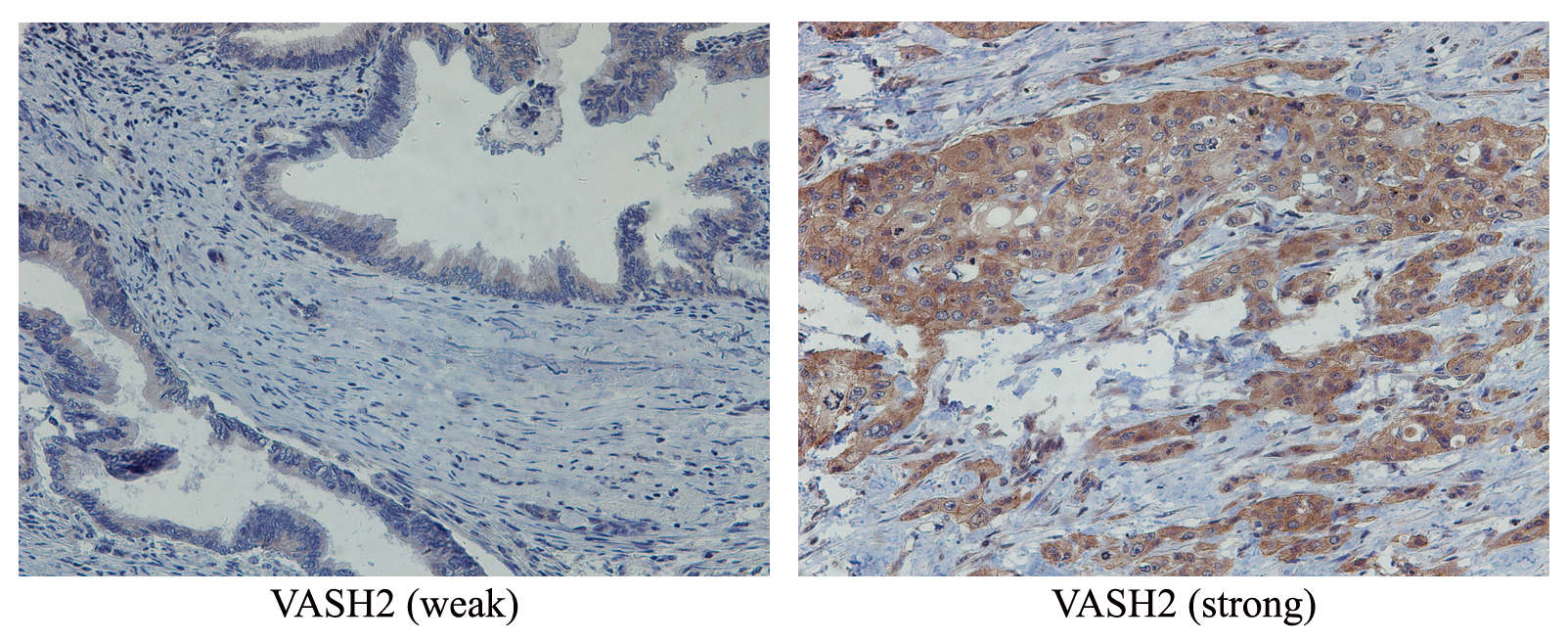

Supplement: Additional file 2: — Representation of immunostaining pictures for weak and strong VASH2 staining in pancreatic cancer tissues. (TIF 3090 kb) [file 12943_2017_619_MOESM2_ESM.tif]

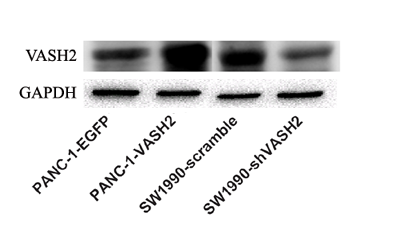

Supplement: Additional file 3: — Expression of VASH2 in stably transfected PANC-1 and SW1990 pancreatic cancer cells. PANC-1 cells were transfected with a vector expressing EGFP (PANC-1-EGFP) or VASH2 (PANC-1-VASH2), and SW1990 cells were transduced with a scrambled shRNA (SW1990-scramble) or a shRNA targeting VASH2 (SW1990-shVASH2). VASH2 protein expression was assessed by immunoblotting. (TIF 294 kb) [file 12943_2017_619_MOESM3_ESM.tif]

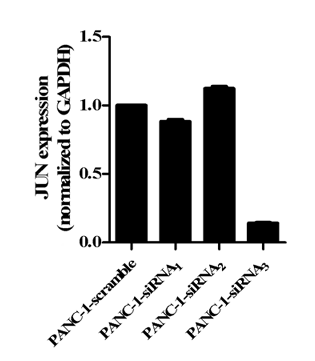

Supplement: Additional file 5: — mRNA expression of JUN in PANC-1 pancreatic cancer cells transfected with different JUN-targeting siRNA. (TIF 454 kb) [file 12943_2017_619_MOESM5_ESM.tif]
